# Hematological impact of COVID-19 mRNA vaccination in patients treated with Clozapine

**DOI:** 10.1192/j.eurpsy.2024.1070

**Published:** 2024-08-27

**Authors:** A. Touiti, I. Yaich, C. Ben Said, N. Bram

**Affiliations:** ^1^Forensic Psychiatry Departement, Razi Hospital, La Manouba; ^2^Faculty of Medecine of Tunis, Tunis El Manar University, Tunis, Tunisia

## Abstract

**Introduction:**

Clozapine is an atypical antipsychotic that is primarily prescribed for treatment-resistant schizophrenia. Despite its proven efficacy, the prescription of clozapine is sometimes limited by its hematologic side effects, including agranulocytosis. During the SARS-CoV-2 pandemic, schizophrenia is recognized as a risk factor for developing severe forms of the infection. Early and complete vaccination of patients has been recommended. However, there is limited data on the effect of the vaccine on the risk of hematologic abnormalities in patients treated with clozapine.

**Objectives:**

To study the hematologic effect of the mRNA vaccine against COVID-19 in a population of patients with treatment-resistant schizophrenia treated with clozapine.

**Methods:**

Twenty-five patients hospitalized for schizophrenia at the forensic psychiatry department of Razi Hospital in Manouba, Tunisia, were included. Eighteen patients were treated with clozapine, and seven patients were treated with other antipsychotics. Consent from patients and/or their relatives was obtained before vaccination. The results of complete blood counts performed as part of the therapeutic protocol were compared between the two groups before and after administration of the vaccine.

**Results:**

No patient experienced agranulocytosis induced by clozapine after vaccination against COVID-19.Blood cells counts,red blood cells counts,and platelets were within the normal ranges.However,a decrease in the number of WBCs,neutrophils,and lymphocytes was observed in patients treated with clozapine without significant difference compared to the patients treated with other neuroleptics, but there was no severe neutropenia or need to stop treatment.

**Conclusions:**

The prescription of clozapine, the introduction protocol, and treatment administration have been greatly influenced by the COVID-19 pandemic due to the hepatotoxic risk of the drug. Vaccination is essential to prevent severe forms of the infection, especially in at-risk populations such as patients treated for schizophrenia.The potentiation of hematologic side effects induced by clozapine by the vaccine is not documented.The COVID-19 mRNA vaccine is safe even with clozapine

**Disclosure of Interest:**

None Declared

